# Supplementary material for: Multimodal analysis of cell-free DNA whole-methylome sequencing for cancer detection and localization
Source: Nat Commun. 2023 Sep 27;14:6042. doi: 10.1038/s41467-023-41774-w (PMC10533817; doi:10.1038/s41467-023-41774-w)
Supplement: Supplementary file 1 — Supplementary Information [file 41467_2023_41774_MOESM1_ESM.pdf]

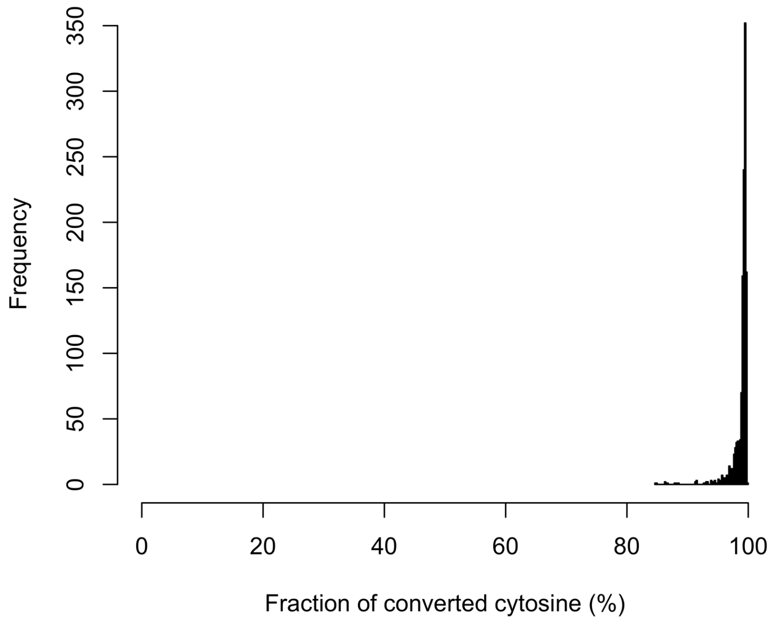

Supplementary Figure S1. Conversion rates of unmethylated cytosine. Histogram depicting the distribution of conversion rates of unmethylated cytosine for the cfDNA WMS data from the MONITOR cohort (*n*=1,277 samples). Source data are provided as a Source Data file.

a

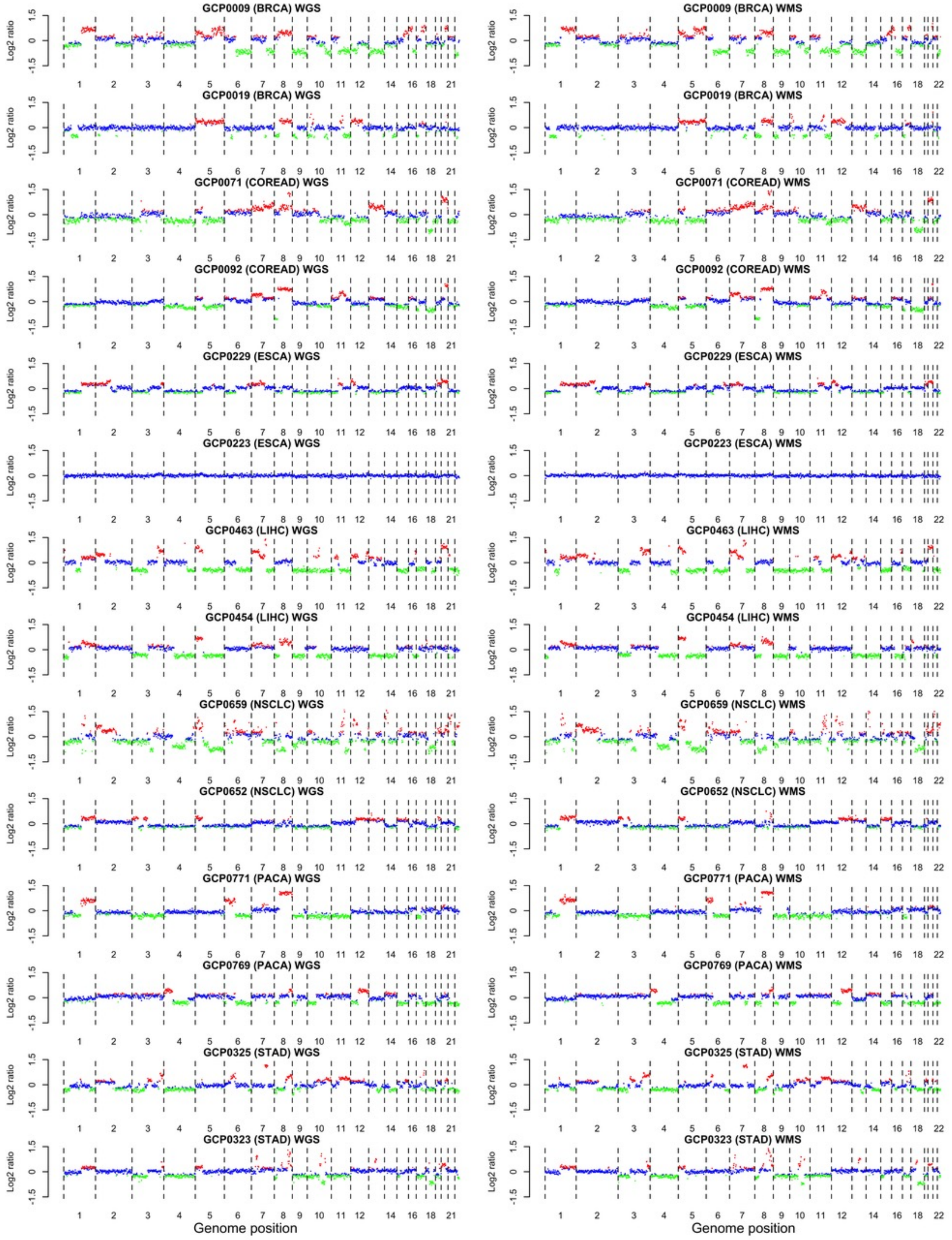
GCP0009 (BRCA) WGS
GCP0009 (BRCA) WMS
GCP0019 (BRCA) WGS
GCP0019 (BRCA) WMS
GCP0071 (COREAD) WGS
GCP0071 (COREAD) WMS
GCP0092 (COREAD) WGS
GCP0092 (COREAD) WMS
GCP0229 (ESCA) WGS
GCP0229 (ESCA) WMS
GCP0223 (ESCA) WGS
GCP0223 (ESCA) WMS
GCP0463 (LIHC) WGS
GCP0463 (LIHC) WMS
GCP0454 (LIHC) WGS
GCP0454 (LIHC) WMS
GCP0659 (NSCLC) WGS
GCP0659 (NSCLC) WMS
GCP0652 (NSCLC) WGS
GCP0652 (NSCLC) WMS
GCP0771 (PACA) WGS
GCP0771 (PACA) WMS
GCP0769 (PACA) WGS
GCP0769 (PACA) WMS
GCP0325 (STAD) WGS
GCP0325 (STAD) WMS
GCP0323 (STAD) WGS
GCP0323 (STAD) WMS
Log2 ratio
Genome position

Cont.

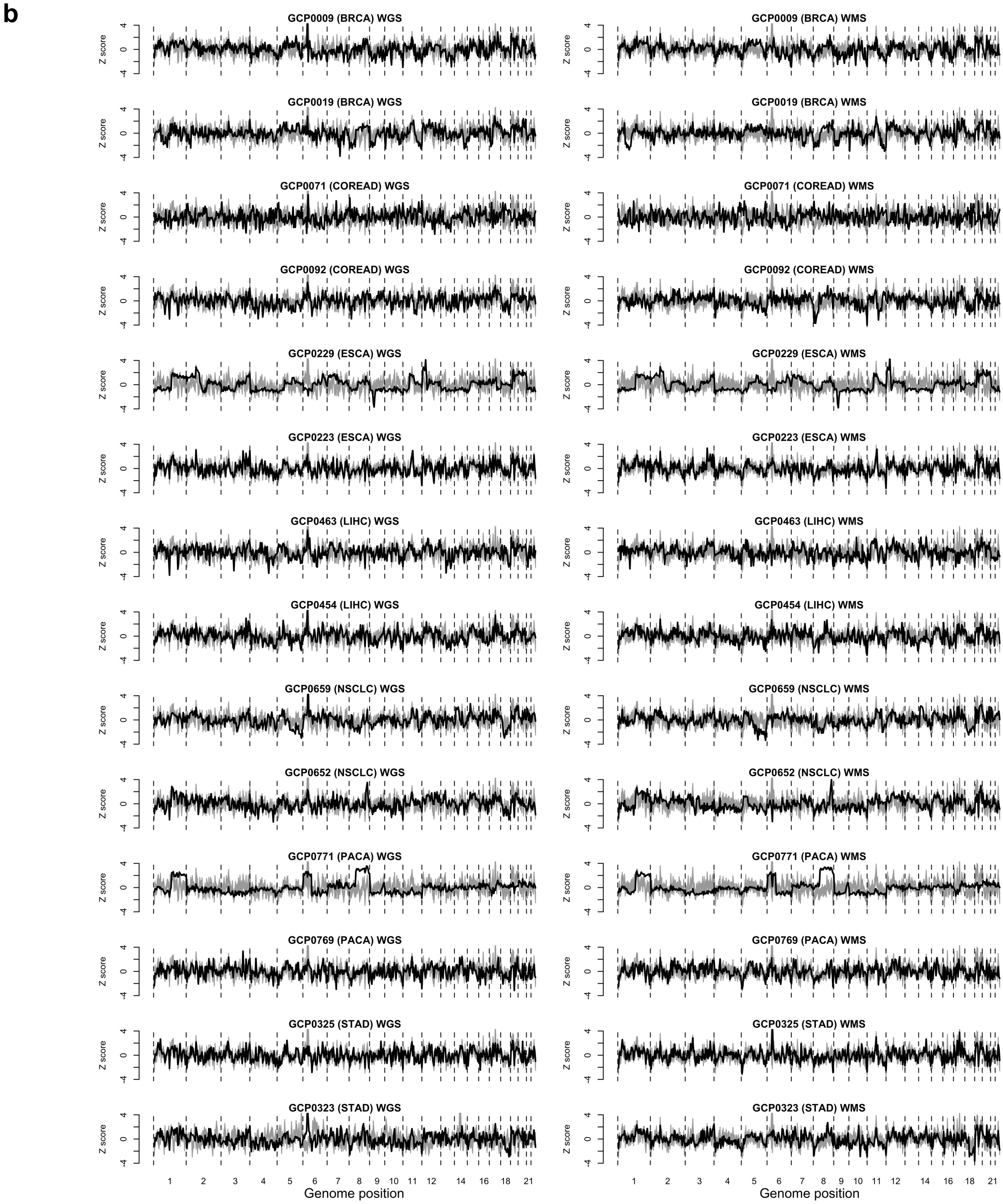

Supplementary Figure S2. Concordance between WMS and WGS CNA and FSI profiling. Two samples from each of the seven cancer types are shown. **(a)** Log2 ratio of coverage over the mean coverage of 225 healthy controls in 100-kb bins across the genome profiled by WGS and WMS respectively. Bins with log2 ratio above 0.3 are colored in red, and bins with log2 ratio below -0.3 are colored in green. **(b)** FSI of 5-Mb windows across the genome profiled by WGS and WMS respectively. The coverage ratio of short to long fragments for each bin is normalized by z-score across the genome. Patient FSI profile is colored in black against 225 grey healthy reference FSI profiles. Source data are provided as a Source Data file.

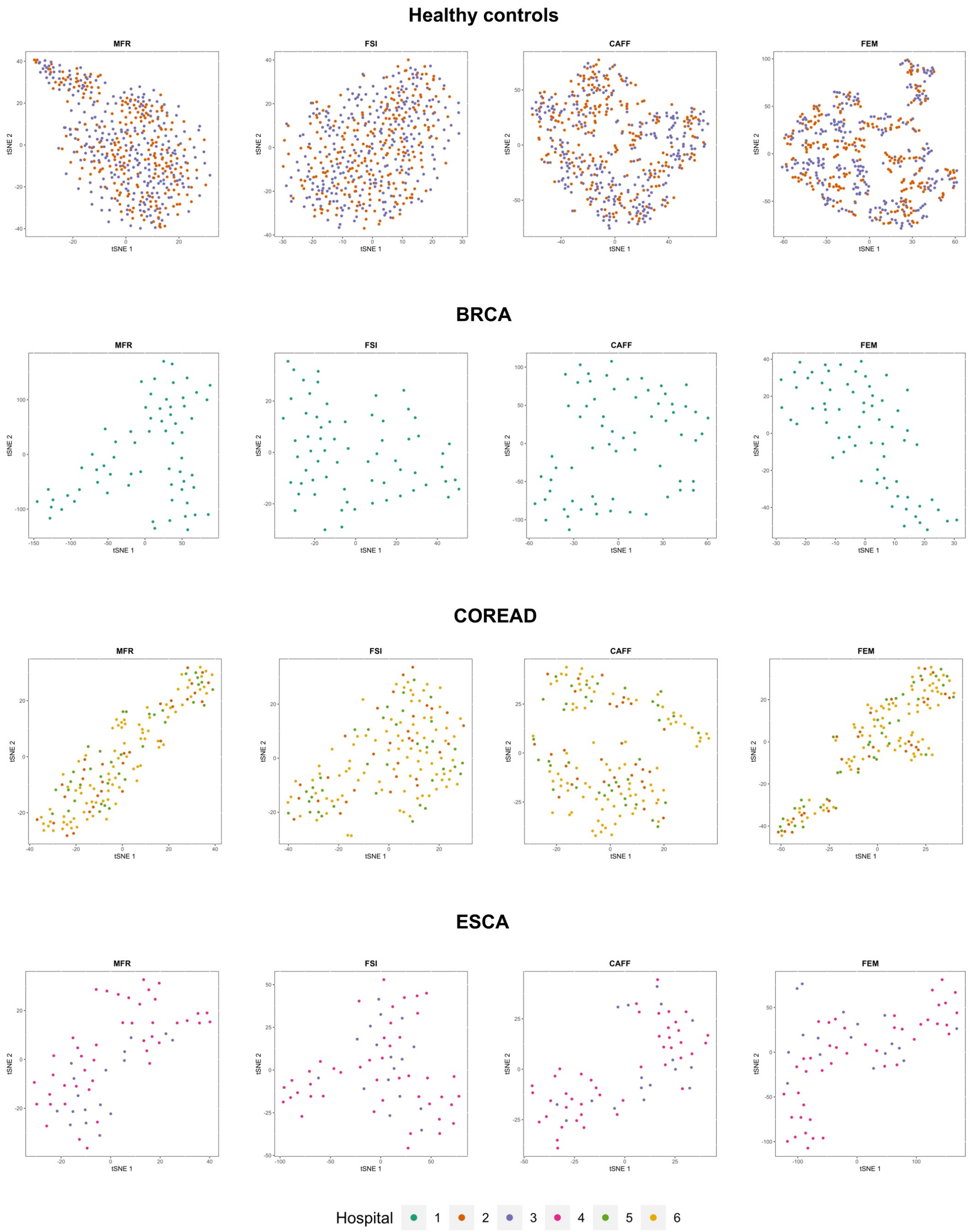
Healthy controls
BRCA
COREAD
ESCA
MFR
FSI
CAFF
FEM
tSNE 1
tSNE 2
Hospital
1
2
3
4
5
6

Cont.

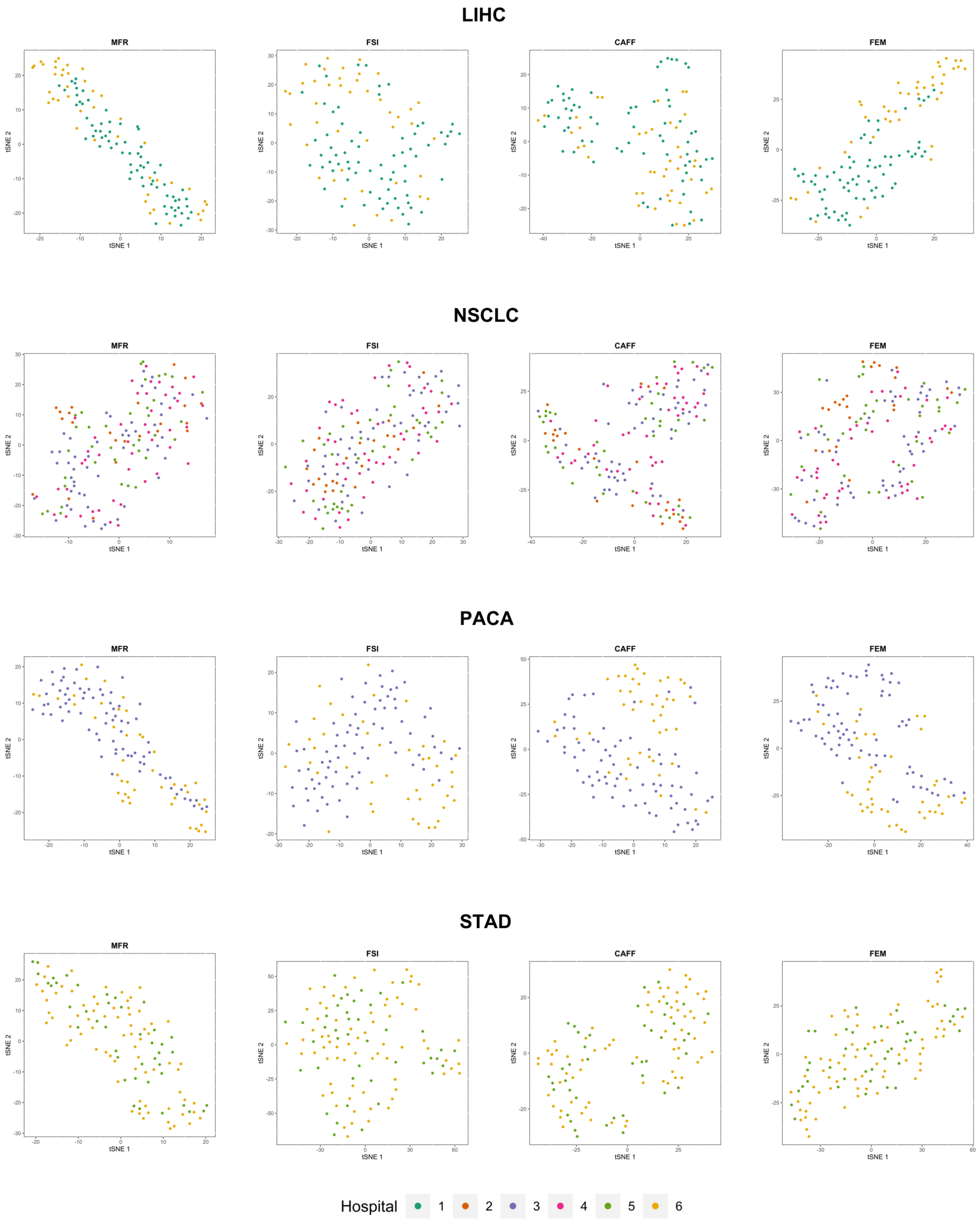

Supplementary Figure S3. tSNE plots of individual features for healthy controls and each cancer type. Hospital source is indicated by color (*n*: Healthy=497, BRCA=66, COREAD=150, ESCA=61, LIHC=113, NSCLC=157, PACA=119, STAD=114). Source data are provided as a Source Data file.

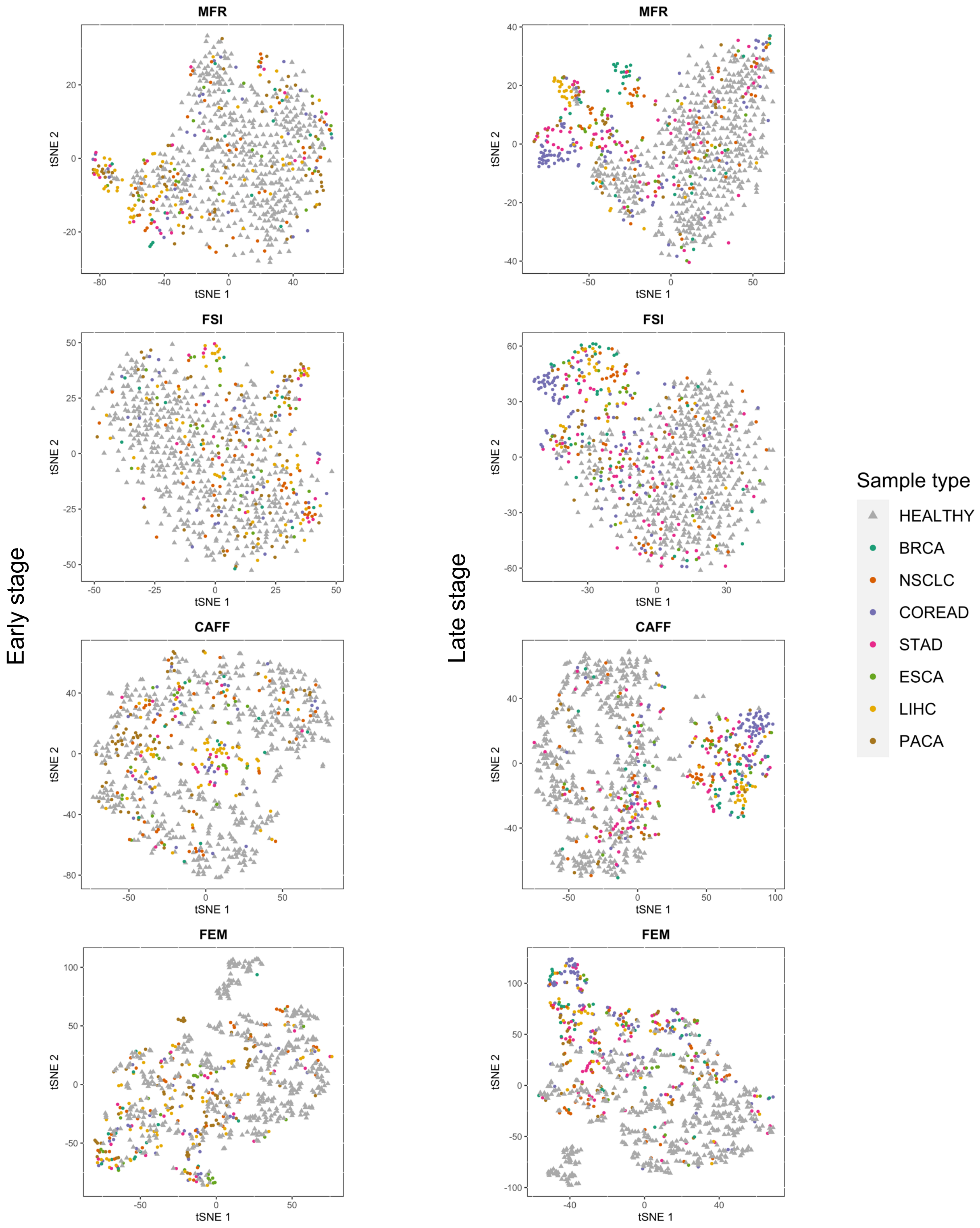

Supplementary Figure S4. tSNE plots of individual features. Early-stage (I and II, n=272) and late-stage (III and IV, n=375) cancer samples are plotted separately. Healthy controls (n=497) and patient samples from different cancer types are annotated by shapes and colors. Source data are provided as a Source Data file.

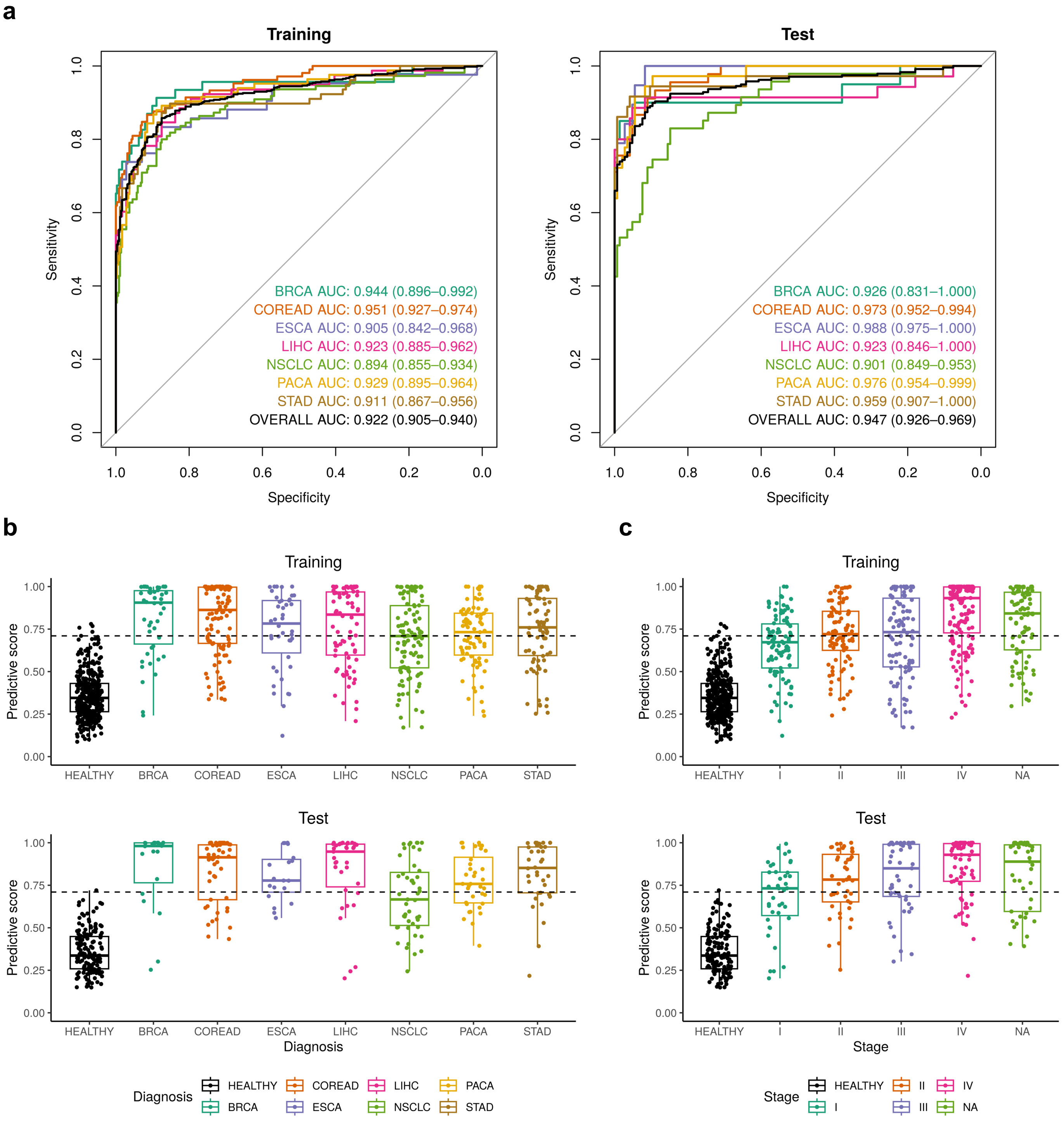

Supplementary Figure S5. Cancer detection performance of MFR. (a) ROC curves for the detection of seven types of cancer using MFR (Training *n* : Healthy=352, BRCA=46, COREAD=105, ESCA=42, LIHC=78, NSCLC=110, PACA=83, STAD=78; Test *n* : Healthy= 145, BRCA=20, COREAD=45, ESCA=19, LIHC=35, NSCLC=47, PACA=36, STAD=36). **(b)** Predictive scores of MFR by cancer type. **(c)** Predictive scores of MFR by histological stage (Training *n* : Healthy=352, stage I=97, stage II=93, stage III=102, stage IV=158, stage NA=92; Test *n* : Healthy=145, stage I=39, stage II=43, stage III=47, stage IV=68, stage NA=41). The dotted line indicates the threshold (positive or negative) at 99% training specificity. The center line in the boxplots represents the median, the lower and upper limits of the box represent the first and third quartiles, and the lower and upper whiskers represent the minimum and maximum values of the data within 1.5 times the inter-quartile range from the lower and upper box limits respectively. Source data are provided as a Source Data file.

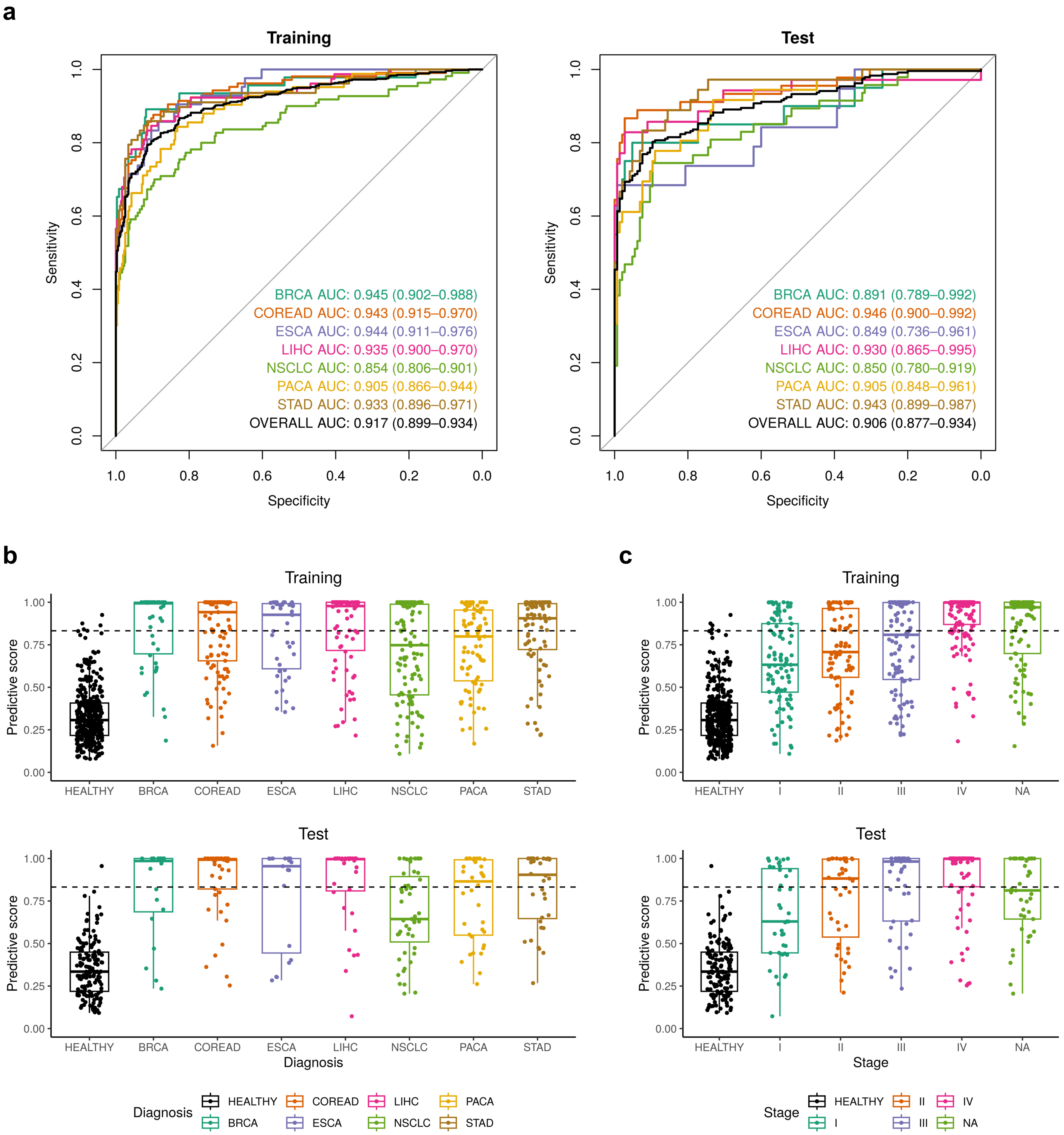

Supplementary Figure S6. Cancer detection performance of FSI. (a) ROC curves for the detection of seven types of cancer using FSI (Training *n* : Healthy=352, BRCA=46, COREAD=105, ESCA=42, LIHC=78, NSCLC=110, PACA=83, STAD=78; Test *n* : Healthy= 145, BRCA=20, COREAD=45, ESCA=19, LIHC=35, NSCLC=47, PACA=36, STAD=36). **(b)** Predictive scores of FSI by cancer type. **(c)** Predictive scores of FSI by histological stage (Training *n* : Healthy=352, stage I=97, stage II=93, stage III=102, stage IV=158, stage NA=92; Test *n* : Healthy=145, stage I=39, stage II=43, stage III=47, stage IV=68, stage NA=41). The dotted line indicates the threshold (positive or negative) at 99% training specificity. The center line in the boxplots represents the median, the lower and upper limits of the box represent the first and third quartiles, and the lower and upper whiskers represent the minimum and maximum values of the data within 1.5 times the inter-quartile range from the lower and upper box limits respectively. Source data are provided as a Source Data file.

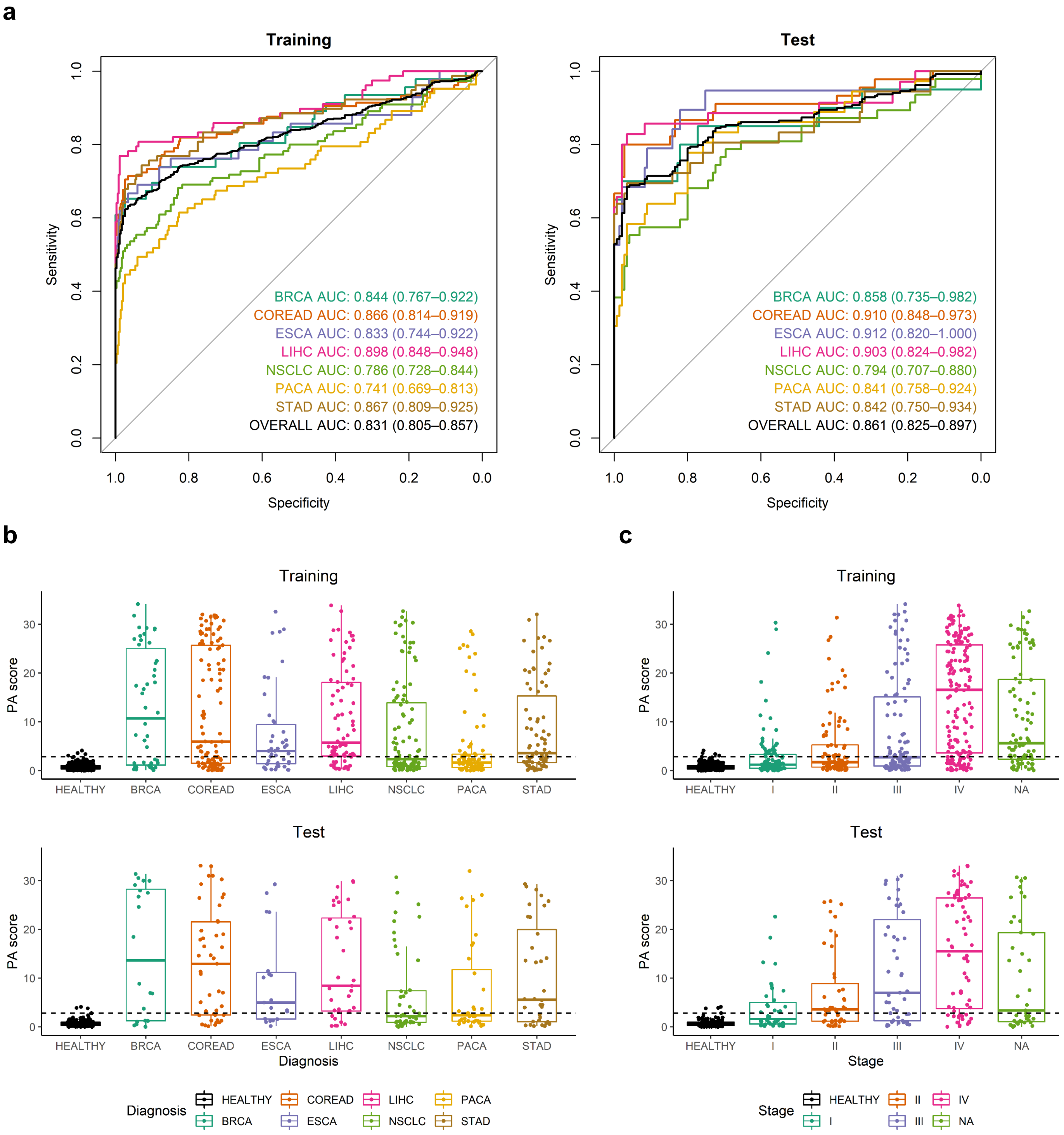

Supplementary Figure S7. Cancer detection performance of CAFF. (a) ROC curves for the detection of seven types of cancer using PA scores of CAFF (Training *n* : Healthy=352, BRCA=46, COREAD=105, ESCA=42, LIHC=78, NSCLC=110, PACA=83, STAD=78; Test *n* : Healthy= 145, BRCA=20, COREAD=45, ESCA=19, LIHC=35, NSCLC=47, PACA=36, STAD=36). **(b)** PA scores of CAFF by cancer type. **(c)** PA scores of CAFF by histological stage (Training *n* : Healthy=352, stage I=97, stage II=93, stage III=102, stage IV=158, stage NA=92; Test *n* : Healthy=145, stage I=39, stage II=43, stage III=47, stage IV=68, stage NA=41). The dotted line indicates the threshold (positive or negative) at 99% training specificity. The center line in the boxplots represents the median, the lower and upper limits of the box represent the first and third quartiles, and the lower and upper whiskers represent the minimum and maximum values of the data within 1.5 times the inter-quartile range from the lower and upper box limits respectively. Source data are provided as a Source Data file.

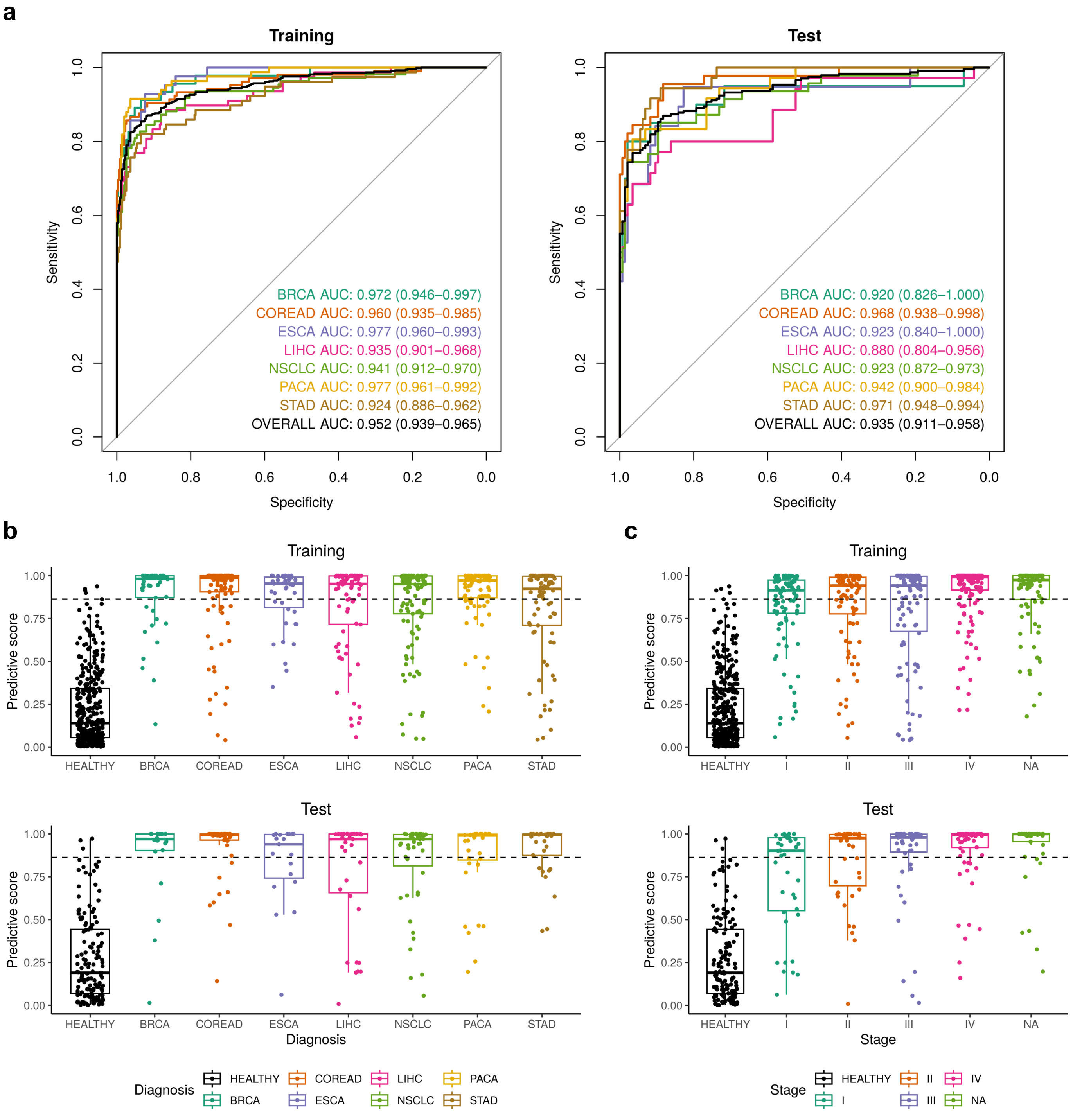

Supplementary Figure S8. Cancer detection performance of FEM. (a) ROC curves for the detection of seven types of cancer using FEM (Training *n* : Healthy=352, BRCA=46, COREAD=105, ESCA=42, LIHC=78, NSCLC=110, PACA=83, STAD=78; Test *n* : Healthy= 145, BRCA=20, COREAD=45, ESCA=19, LIHC=35, NSCLC=47, PACA=36, STAD=36). **(b)** Predictive scores of FEM by cancer type. **(c)** Predictive scores of FEM by histological stage (Training *n* : Healthy=352, stage I=97, stage II=93, stage III=102, stage IV=158, stage NA=92; Test *n* : Healthy=145, stage I=39, stage II=43, stage III=47, stage IV=68, stage NA=41). The dotted line indicates the threshold (positive or negative) at 99% training specificity. The center line in the boxplots represents the median, the lower and upper limits of the box represent the first and third quartiles, and the lower and upper whiskers represent the minimum and maximum values of the data within 1.5 times the inter-quartile range from the lower and upper box limits respectively. Source data are provided as a Source Data file.

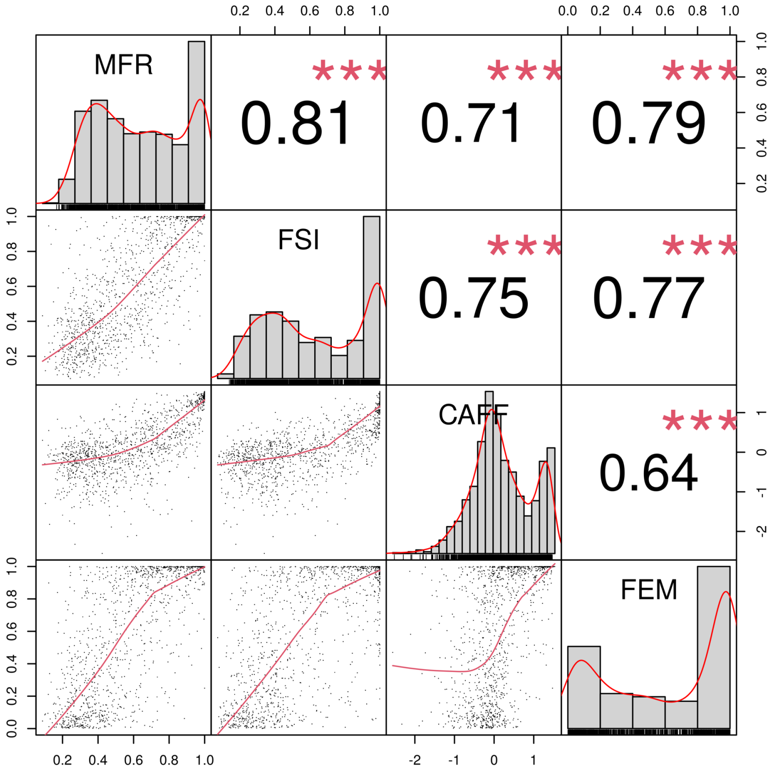

Supplementary Figure S9. Correlation between individual features extracted from WMS data. Histograms depict the distributions of log10 transformed PA scores of CAFF and predictive scores of FSI, MFR, and FEM respectively (n=1,277 samples). Scatter plots depict the pairwise correlations between features and regression lines are fitted. Spearman correlation coefficients are calculated and asterisks (***) denote $p < 0.001$.

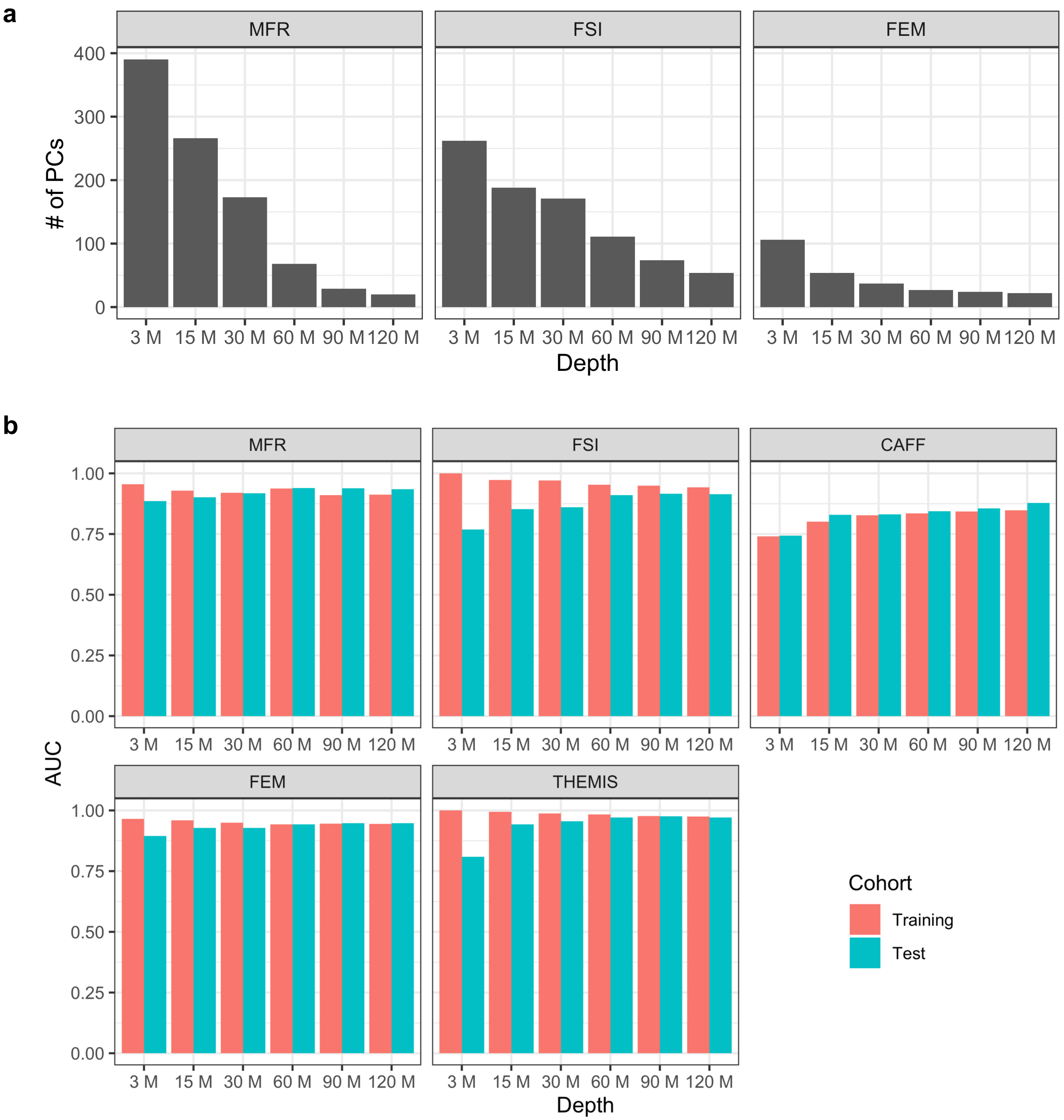

Supplementary Figure S10. Performance of cancer detection with downsampling of sequencing depth. WMS data from 306 healthy controls and 467 cancer patients were randomly subsampled to 120M, 90M, 60M, 30M, 15M, and 3M paired reads respectively. **(a)** The number of top principle components to explain 95% of variance for MFR, 95% of variance for FSI, and 90% variance for FEM. **(b)** AUCs of individual modalities and the THEMIS model at each sequencing depth, split by training and test cohorts. Source data are provided as a Source Data file.

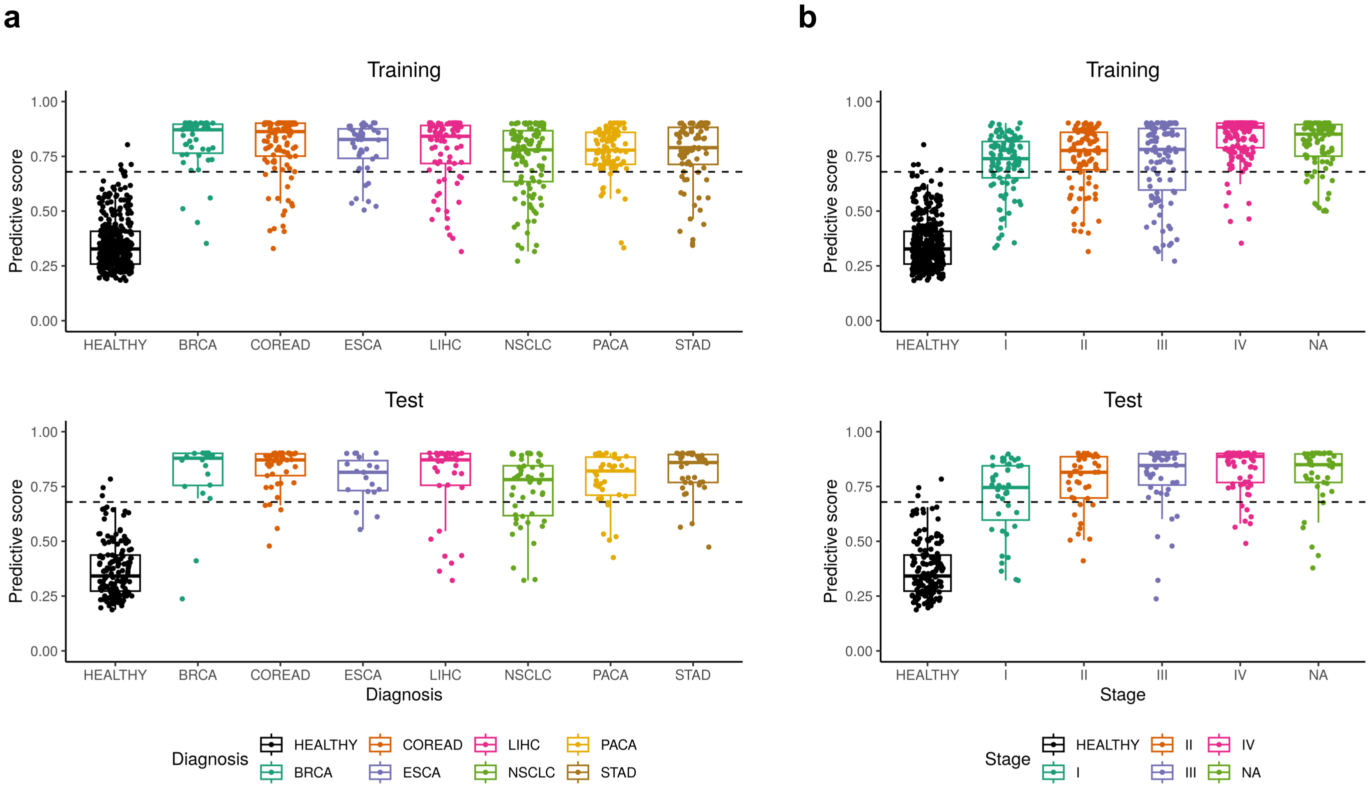

Supplementary Figure S11. THEMIS prediction scores by cancer type and histological stage. (a) Predictive scores of THEMIS by cancer type (Training *n* : Healthy=352, BRCA=46, COREAD=105, ESCA=42, LIHC=78, NSCLC=110, PACA=83, STAD=78; Test *n* : Healthy= 145, BRCA=20, COREAD=45, ESCA=19, LIHC=35, NSCLC=47, PACA=36, STAD=36). **(b)** Predictive scores of THEMIS by histological stage (Training *n* : Healthy=352, stage I=97, stage II=93, stage III=102, stage IV=158, stage NA=92; Test *n* : Healthy=145, stage I=39, stage II=43, stage III=47, stage IV=68, stage NA=41). The dotted line indicates the threshold (positive or negative) at 99% training specificity. The center line in the boxplots represents the median, the lower and upper limits of the box represent the first and third quartiles, and the lower and upper whiskers represent the minimum and maximum values of the data within 1.5 times the inter-quartile range from the lower and upper box limits respectively.

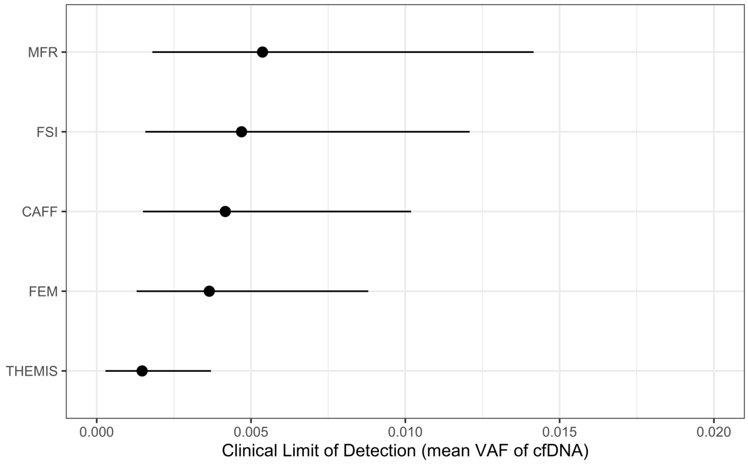

Supplementary Figure S12. Clinical LOD for each classifier. The clinical LOD was measured as the mean VAF corresponding to 50% cancer signal detection at 99% specificity for individual modalities and the integrative THEMIS model, using *n*=65 samples whose paired tumor tissue and WBC samples were available. Error bars indicate 95% confidence intervals. Source data are provided as a Source Data file.

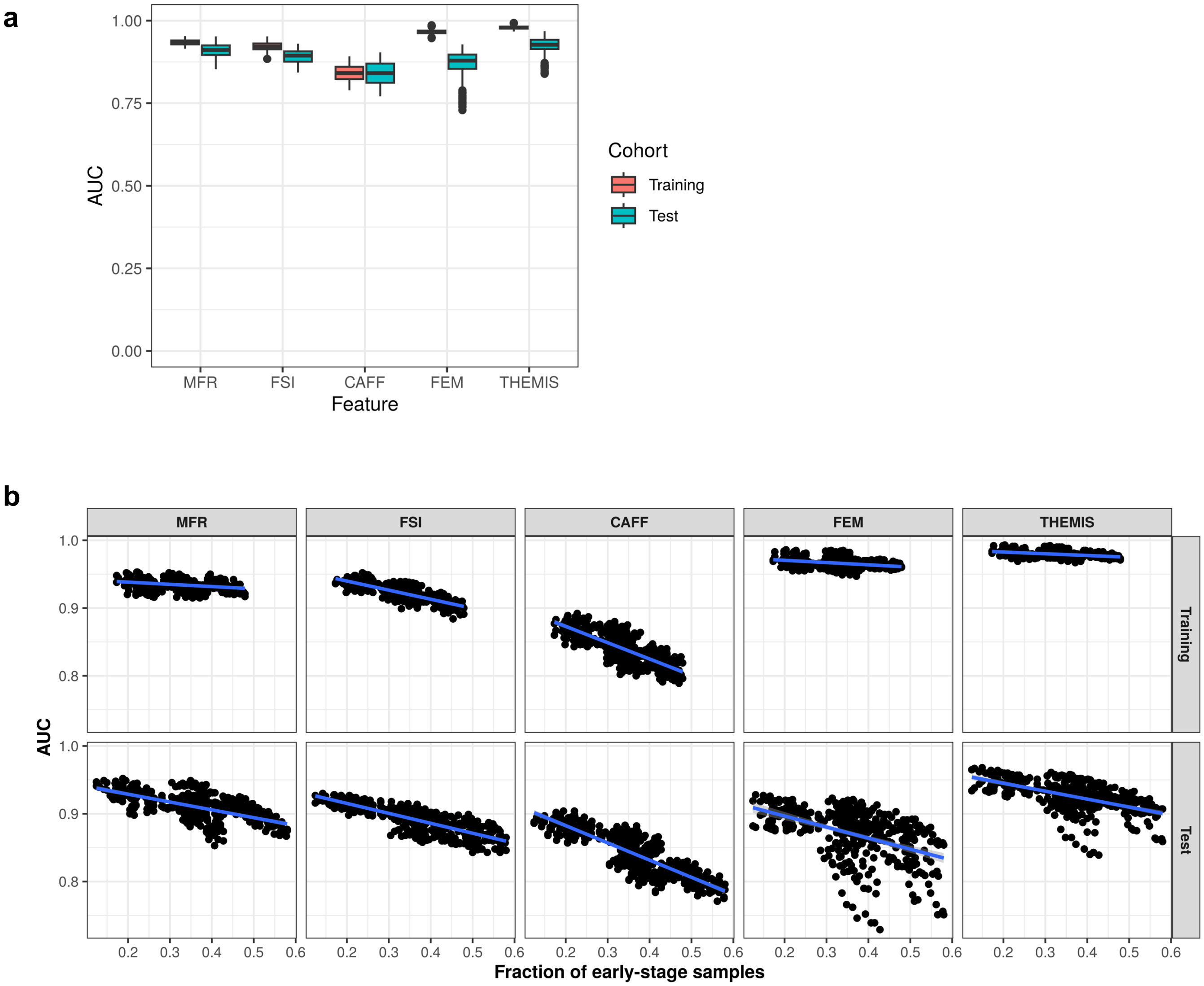

Supplementary Figure S13. Evaluation of classifier robustness to sample collection source. A total of *n*=384 split-by-hospital combinations are analyzed. **(a)** AUCs of individual modalities and the THEMIS model for each combination, split by training and test cohorts. The center line in the boxplots represents the median, the lower and upper limits of the box represent the first and third quartiles, the lower and upper whiskers represent the minimum and maximum values of the data within 1.5 times the inter-quartile range from the lower and upper box limits respectively, and individual points represent outlier data beyond the end of the whiskers. **(b)** Scatter plots of model AUCs of individual modalities and THEMIS versus the proportion of early-stage (I or II) cancer samples for each combination, split by training and test cohorts. Source data are provided as a Source Data file.

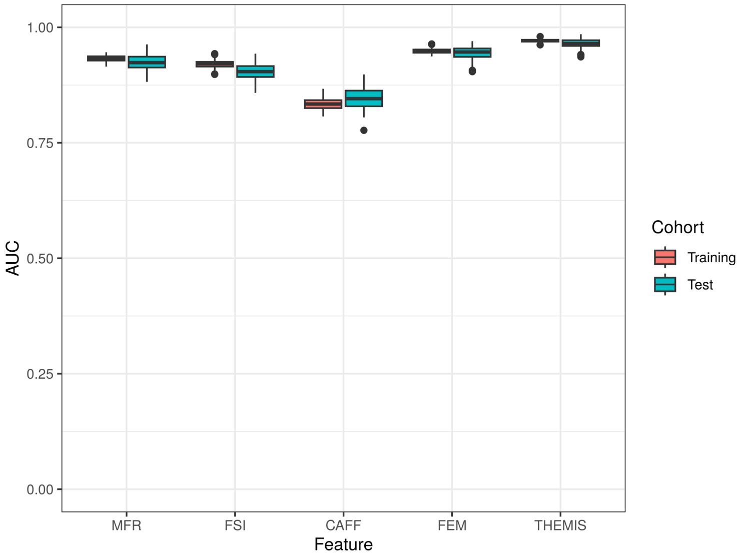

Supplementary Figure S14. Classifier performances over multiple random splits of the dataset. Box plots depicting AUCs of individual modalities and the integrative THEMIS model over *n*=100 runs are shown. The center line in the boxplots represents the median, the lower and upper limits of the box represent the first and third quartiles, the lower and upper whiskers represent the minimum and maximum values of the data within 1.5 times the inter-quartile range from the lower and upper box limits respectively, and individual points represent outlier data beyond the end of the whiskers. Source data are provided as a Source Data file.

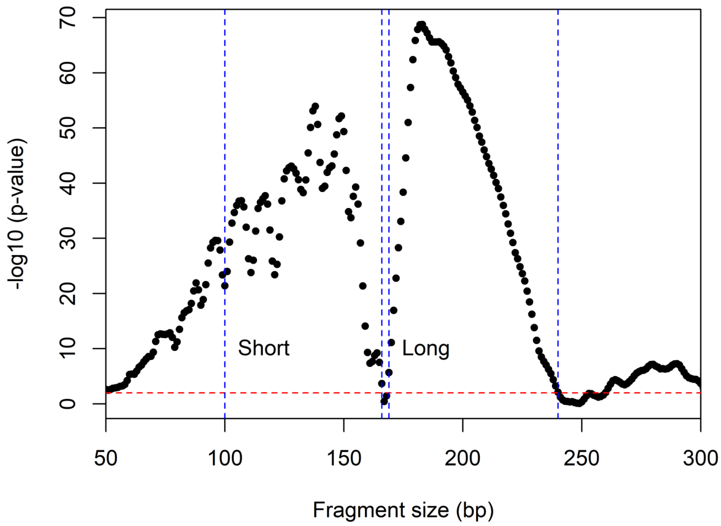

Supplementary Figure S15. Difference in cfDNA size distribution between cancer patients and healthy controls. Wilcoxon one-sided test is performed at each fragment size between the frequencies of cancer patients (*n*=542) and healthy controls (*n*=352) in the training cohort, and –log10 of p values are plotted against fragment sizes. The red dotted line indicates the threshold of significance, p values above which (0.01) are considered insignificant. Blue dotted lines mark the size boundaries of short (100–166 bp) and long (169–240 bp) fragments. Source data are provided as a Source Data file.

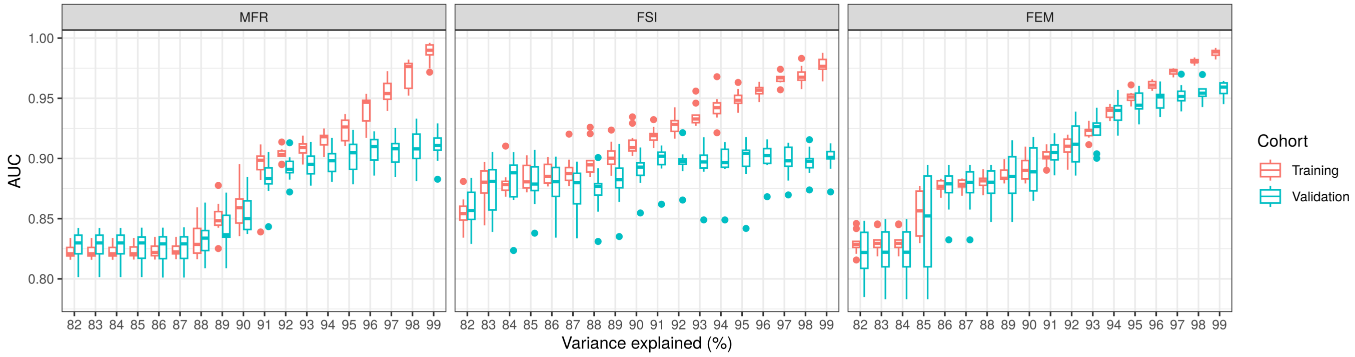

Supplementary Figure S16. Model performances for the training and validation folds for MFR, FSI, and FEM modalities. Box plots summarizing model AUCs of the training and validation folds of the training cohort over *n*=10 bootstraps are plotted against data variance explained by selected principle components. The center line in the boxplots represents the median, the lower and upper limits of the box represent the first and third quartiles, the lower and upper whiskers represent the minimum and maximum values of the data within 1.5 times the inter-quartile range from the lower and upper box limits respectively, and individual points represent outlier data beyond the end of the whiskers. Source data are provided as a Source Data file.
