# Supplementary material for: Multimodal analysis of cell-free DNA whole-methylome sequencing for cancer detection and localization
Source: Nat Commun. 2023 Sep 27;14:6042. doi: 10.1038/s41467-023-41774-w (PMC10533817; doi:10.1038/s41467-023-41774-w)
Supplement: Supplementary file 3 — Reporting Summary [file 41467_2023_41774_MOESM3_ESM.pdf]

nature portfolio

Corresponding author(s): Fengwei Tan, Shugeng Gao

Last updated by author(s): Aug 21, 2023

Reporting Summary

Nature Portfolio wishes to improve the reproducibility of the work that we publish. This form provides structure for consistency and transparency in reporting. For further information on Nature Portfolio policies, see our Editorial Policies and the Editorial Policy Checklist.

Statistics

For all statistical analyses, confirm that the following items are present in the figure legend, table legend, main text, or Methods section.

n/a	Confirmed	
☐	☒	The exact sample size (*n*) for each experimental group/condition, given as a discrete number and unit of measurement
☐	☒	A statement on whether measurements were taken from distinct samples or whether the same sample was measured repeatedly
☐	☒	The statistical test(s) used AND whether they are one- or two-sided<br>*Only common tests should be described solely by name; describe more complex techniques in the Methods section.*
☐	☒	A description of all covariates tested
☐	☒	A description of any assumptions or corrections, such as tests of normality and adjustment for multiple comparisons
☐	☒	A full description of the statistical parameters including central tendency (e.g. means) or other basic estimates (e.g. regression coefficient) AND variation (e.g. standard deviation) or associated estimates of uncertainty (e.g. confidence intervals)
☐	☒	For null hypothesis testing, the test statistic (e.g. *F*, *t*, *r*) with confidence intervals, effect sizes, degrees of freedom and *P* value noted<br>*Give P values as exact values whenever suitable.*
☒	☐	For Bayesian analysis, information on the choice of priors and Markov chain Monte Carlo settings
☒	☐	For hierarchical and complex designs, identification of the appropriate level for tests and full reporting of outcomes
☐	☒	Estimates of effect sizes (e.g. Cohen's *d*, Pearson's *r*), indicating how they were calculated

Our web collection on statistics for biologists contains articles on many of the points above.

Software and code

Policy information about availability of computer code

Data collection	No software was used for data collection.
Data analysis	bcl2fastq(v2.20.0), Trimmomatic (v0.36),BisMark (v0.19.0), samtools (v1.3), BamUtil(v1.0.14), Python (v3.7.3), sklearn (v1.2.0), R (v4.0.2).<br><br>Custom codes and scripts are publicly available at https://github.com/yulongbio/themis.git

For manuscripts utilizing custom algorithms or software that are central to the research but not yet described in published literature, software must be made available to editors and reviewers. We strongly encourage code deposition in a community repository (e.g. GitHub). See the Nature Portfolio guidelines for submitting code & software for further information.

Data

Policy information about availability of data

All manuscripts must include a data availability statement. This statement should provide the following information, where applicable:

- Accession codes, unique identifiers, or web links for publicly available datasets
- A description of any restrictions on data availability
- For clinical datasets or third party data, please ensure that the statement adheres to our policy

The 1277 cfDNA WMS data of the MONITOR cohort generated in this study have been deposited in the Genome Sequence Archive (GSA) for Human database under accession code HRA003209 [https://ngdc.cncb.ac.cn/gsa-human/browse/HRA003209]. To protect patient privacy, data access can be obtained through a request to

the data access committee. Access to the data will be restricted to non-commercial entities. Access will be provided within approximately one week and be available for one year. Tissue ATAC-seq peaks were download from TCGA [https://gdc.cancer.gov/about-data/publications/ATACseq-AWG]. Reference genome hg19 was used for mapping samples. Source data are provided with this paper.

Research involving human participants, their data, or biological material

Policy information about studies with human participants or human data. See also policy information about sex, gender (identity/presentation), and sexual orientation and race, ethnicity and racism.

Reporting on sex and gender	Analyses performed in this study are based on cancer types and disease stages. Sex- or gender-based analyses are not involved.
Reporting on race, ethnicity, or other socially relevant groupings	Analyses performed in this study are based on cancer types and disease stages. Race, ethnicity, or other socially revevant groupings are not involved.
Population characteristics	Human plasma samples are obtained from six medical centers affiliated with National Cancer Center, including the Cancer Hospital Chinese Academy of Medical Sciences, Zhongshan Hospital Fudan University, Ruijin Hospital Shanghai Jiaotong University School of Medicine, The First Affiliated Hospital of Soochow University, Beijing Cancer Hospital, and Shanxi Provincial Cancer Hospital. The participants are socioeconomically and regionally diverse. Detailed population information is provided in Supplementary Table S1.
Recruitment	Participant age must be at least 18. Cancer type and stage are diagnosed by histological or cytological evaluation and blood samples are obtained before tumor resection or therapy. Individuals showing no symptom of cancer and had no prior history of cancer were considered as healthy controls. Pregnant participants are also excluded.
Ethics oversight	The ethics committee of National Cancer Center approved the protocol of this study (NCC–007821), and our research complies with all relevant ethical regulations. All participants gave their written informed consent for research use.

Note that full information on the approval of the study protocol must also be provided in the manuscript.

Field-specific reporting

Please select the one below that is the best fit for your research. If you are not sure, read the appropriate sections before making your selection.

☒ Life sciences ☐ Behavioural & social sciences ☐ Ecological, evolutionary & environmental sciences

For a reference copy of the document with all sections, see nature.com/documents/nr-reporting-summary-flat.pdf

Life sciences study design

All studies must disclose on these points even when the disclosure is negative.

Sample size	We examined plasma samples collected from healthy controls (n = 497) and cancer patients (n = 780), including breast (n = 66), colorectal (n = 150), esophageal (n = 61), liver (n = 113), lung (n = 157), pancreatic (n = 119), and gastric (n = 114) cancers. An analysis of approximately 800 patients with cancer and 500 healthy controls suffices for estimation of a sensitivity of 0.8 with a margin of error of 0.05 at a specificity of 0.95.
Data exclusions	No data were excluded from the analyses.
Replication	We confirmed the technical reproducibility of WMS assay with 10 internal pilot samples, all of which showed highly similar results between two technical replicates. Clinical samples of the MONITOR cohort analyzed in this study had no WMS replicates given the limited amount of blood sampled from participants. The concordance between WMS and WGS assays in FSI and CNA profiling are confirmed with a subset of MONITOR samples (n=490, see Table S2), as described in Results.<br>The reproducibility of modeling performance was confirmed by 100 random training/testing splits.
Randomization	The experiments were not randomized because this is a retrospective case-control study. For the development of the classifier, the training and testing cohorts were randomly split and balanced by age, sex, and cancer type/stage. During model training, we performed 10 bootstraps wherein each bootstrap randomly selected 70% of the training cohort for model training and the remaining 30% for model validation. The average probability of the resulting 10 sub-models was used as the final prediction score of a sample.
Blinding	Not applicable to this study. We designed a retrospective study to develop a classifier for cancer screening, and knowledge of the health status and cancer types/stages was necessary to evaluate the methods.

Reporting for specific materials, systems and methods

We require information from authors about some types of materials, experimental systems and methods used in many studies. Here, indicate whether each material, system or method listed is relevant to your study. If you are not sure if a list item applies to your research, read the appropriate section before selecting a response.

Materials & experimental systems

n/a	Involved in the study
☒	☐ Antibodies
☒	☐ Eukaryotic cell lines
☒	☐ Palaeontology and archaeology
☒	☐ Animals and other organisms
☐	☒ Clinical data
☒	☐ Dual use research of concern
☒	☐ Plants

Methods

n/a	Involved in the study
☒	☐ ChIP-seq
☒	☐ Flow cytometry
☒	☐ MRI-based neuroimaging

Clinical data

Policy information about clinical studies

All manuscripts should comply with the ICMJE guidelines for publication of clinical research and a completed CONSORT checklist must be included with all submissions.

Clinical trial registration	Not applicable for this retrospectively analyzed study.
Study protocol	The protocols are available for research purposes from the corresponding authors on reasonable request.
Data collection	The study was conducted in six hospitals: the Cancer Hospital Chinese Academy of Medical Sciences, Zhongshan Hospital Fudan University, Ruijin Hospital Shanghai Jiaotong University School of Medicine, The First Affiliated Hospital of Soochow University, Beijing Cancer Hospital, and Shanxi Provincial Cancer Hospital. Blood samples were taken from October 2021 to December 2022.
Outcomes	Not applicable for this retrospectively analyzed study.
