# Supplementary material for: Multimodal analysis of cell-free DNA whole-methylome sequencing for cancer detection and localization
Source: Nat Commun. 2023 Sep 27;14:6042. doi: 10.1038/s41467-023-41774-w (PMC10533817; doi:10.1038/s41467-023-41774-w)
Supplement: Supplementary file 4 — Description of additional supplementary files [file 41467_2023_41774_MOESM4_ESM.pdf]

Description of Additional Supplementary Files Document

File Name: Supplementary Data 1

Description: Clinical information of MONITOR patients analyzed

File Name: Supplementary Data 2

Description: Comparison between WMS and WGS

File Name: Supplementary Data 3

Description: Samples for association analysis among MFR, FSI, and CAN

File Name: Supplementary Data 4

Description: Genomic regions for MFR analysis, hg19 coordinates

File Name: Supplementary Data 5

Description: Genomic regions for FSI analysis, hg19 coordinates

File Name: Supplementary Data 6

Description: Cancer prediction scores by individual modalities and THEMIS for MONITOR patients
